# Comparative Expression Profile Analysis of Apoptosis-Related miRNA and Its Target Gene in *Leishmania* major Infected Macrophages

**DOI:** 10.18502/ijpa.v15i3.4197

**Published:** 2020

**Authors:** Zohreh LASJERDI, Hossein GHANBARIAN, Samira MOHAMMADI YEGANEH, Seyyed Javad SEYYED TABAEI, Mehdi MOHEBALI, Niloofar TAGHIPOUR, Ameneh KOOCHAKI, Faezeh HAMIDI, Mostafa GHOLAMREZAEI, Ali HAGHIGHI

**Affiliations:** 1. Department of Medical Parasitology and Mycology, School of Medicine, Student Research Committee, Shahid Beheshti University of Medical Sciences, Tehran, Iran; 2. Department of Medical Parasitology and Mycology, School of Medicine, Shahid Beheshti University of Medical Sciences, Tehran, Iran; 3. Cellular and Molecular Biology Research Center, Shahid Beheshti University of Medical Sciences, Tehran, Iran; 4. Department of Biotechnology, School of Advanced Technologies in Medicine, Shahid Beheshti University of Medical Sciences, Tehran, Iran; 5. Department of Parasitology and Mycology, School of Public Health, Tehran University of Medical Sciences Tehran, Iran

**Keywords:** Apoptosis, In vitro, *Leishmania major*, Macrophage, MicroRNAs

## Abstract

**Background:**

Cutaneous Leishmaniasis (CL) is an emerging uncontrollable and neglected infectious disease worldwide including Iran. The aim of this study was to investigate the expression profile of apoptosis-related miRNA and its target gene in macrophages.

**Methods:**

This study was carried out in the Department of Medical Parasitology and Mycology, School of Medicine, Shahid Beheshti University of Medical Sciences, Tehran, Iran from January 2016 to November 2018. Applying literature reviews, bioinformatics software, and microarray expression analysis, we selected miRNA-24-3p interfering in apoptosis pathway. The expression profile of this miRNA and target gene were investigated in *Leishmania major* (MRHO/IR/75/ER)-infected primary and RAW 264.7 macrophages (IBRC-C10072) compared with non-infected macrophages (control group) using quantitative Real-time PCR.

**Results:**

Results of bioinformatics analysis showed that miR-24-3p as anti-apoptotic miRNA inhibits pro-apoptotic genes (Caspases 3 and 7). Microarray expression data presented in Gene Expression Omnibus (GEO) revealed a significant difference in the expression level of selected miRNA and its target gene between two groups. QRT-PCR results showed that the expression of miR-24-3p was upregulated in *L. major* infectioned macrophages that approved the results of bioinformatics and microarray analysis.

**Conclusion:**

Parasite can alter miRNAs expression pattern in the host cells to establish infection and its survival. Alteration in miRNAs levels likely plays an important role in regulating macrophage functions following *L. major* infection. These results could highlight current understanding and new insights concerning the gene expression in macrophages during leishmaniasis and will help to development of novel strategies for control and treatment of CL.

## Introduction

Leishmaniasis is a globally disperse disease caused by vector-borne protozoan parasites of the genus *Leishmania* (1). Based on the location of the parasite in the tissues, the various species of parasite cause different forms of clinical manifestations including cutaneous, mucocutaneous, and visceral leishmaniasis (2, 3). More than 12 million people are infected with the parasite, and the annual incidence is 2–2.5 million cases. Cutaneous leishmaniasis (CL) is the most predominant form of the disease (4), and one of the neglected diseases according to WHO (5).

In Iran, CL is one the most important endemic disease in several parts of the country (6–9). Therapeutic intervention is the only effective way of controlling leishmaniasis since there is no effective vaccine for the disease (10, 11). The first choice treatment of CL is pentavalent antimonial compounds particularly meglumine antimonate (Glucantime^®^) and sodium stibogluconate (Pentostam^®^) widely prescribed (12, 13). However, application of therapeutic agents confronts several problems including side effects, high cost, difficulty in administration and drug resistance. Therefore, exploring an effective method for controlling and treatment of the disease seems necessary (11, 14, 15).

The mechanism of anti-leishmanial drugs is parasite or host cell apoptosis induction (16–18). Apoptosis is a form of programmed cell death (PCD) that plays a central role in normal tissue development and organogenesis (19) as well as in the pathogenesis of different diseases (20). PCD also plays an important role in the regulation of the immune response and more generally, in defense against infections (21). Apoptosis is mediated by involving differential expression of specific target genes such as caspase cysteine proteases (22). Moreover, many miRNAs are involved in regulating apoptotic pathways (23).

MicroRNAs, a class of non-coding small RNAs, regulate several biological processes such as cell proliferation, metabolism, differentiation, signaling, and apoptosis. These highly conserved molecules by binding to the 3′-untranslated region (3′-UTR) of their mRNA targets regulate protein expression by degrading target mRNA or inhibiting translation (24, 25). Several miRNAs are dysregulated in Leishmania-infected macrophages when compared with normal cells (26–28). Importantly, due to their significant roles, miRNAs are emerging as therapeutic and diagnostic tools for many diseases such as parasitic diseases (24, 25).

Macrophages and dendritic cells are the most important immune system cells that control the outcome of the infection. Understanding how the parasite maintains its survival is the key element for devising novel therapeutic strategies (29, 30). Since *Leishmania* spp. are intracellular parasites, their survival depends on the manipulation of host cells’ signaling pathways and inhibit macrophages anti-parasitic activity. Moreover, the parasite can alter the miRNAs expression profile in infected cells in favor of its survival (26). To date, several studies described these alternations in human and/or murine macrophages infected with *Leishmania* spp. (26–28, 31).

Considering the important role of apoptosis pathway in parasite survival in macrophages, in this study we attempted to literature review and bioinformatic analysis to identify potential miRNA which target apoptosis genes, and evaluate over-expression or down-regulation of selected miRNA and target gene in primary and cell line macrophages infected with *Leishmania major*, and survey the hypothetical inverse relationship between miRNA and target gene.

## Materials and Methods

### Bioinformatics analysis

Apoptosis pathway regulation in macrophages after *L. major* infection was the target of this study, miRNA prediction and bioinformatic analysis of mRNA:miRNA interaction were performed using various databases such as miRvestigator, miRWalk (http://mirwalk.umm.uni-heidelberg.de/), TargetScan (http://www.targetscan.org/) and DIANA (http://diana.imis.athena-innovation.gr/DianaTools/index.php). Furthermore, the predicted miRNA was further investigated in literature. Finally, we selected miR-24-3p as anti-apoptotic miRNA that regulates intrinsic and extrinsic apoptosis pathways. The study was carried out in Department of Medical Parasitology and Mycology, School of Medicine, Shahid Beheshti University of Medical Sciences, Tehran, Iran from January 2016 to November 2018.

### Animal studies, macrophages isolation and cell culture

Specific pathogen-free female BALB/c mice (6–8 weeks old, 20–25g) as sources of macrophages were purchased from Pasteur institute (Tehran, Iran).

The study protocol and procedures were approved by the Shahid Beheshti University of Medical Science Ethical Committee IR.SBMU.MSP.REC.1396.104 local health authorities. The animals were maintained under standard conditions for at least one week to adaptation. Condition of the animals was monitored during the week and euthanasia was performed by cervical dislocation for animal death.

Primary peritoneal macrophages were isolated from mice in accordance with protocol (32). After centrifuge of the cells in a refrigerated centrifuge 10 min at 400×*g* and count them by hemacytometer, the 8×10^5^ cells were cultured on 6-well plates by using DMEM medium (BIO-IDEA, IRAN) supplemented with 15% (v/v) heat-inactivated fetal bovine serum (FBS, Gibco, USA) and 100U/mL penicillin, 100μg/mL streptomycin (Gibco, USA). The cells incubated at 37 °C with 5% CO2 for 24 h. After 12 h of incubation, cells were washed with DMEM medium twice to remove the non-macrophage cells and the fresh media was added. The murine macrophage cell line (RAW 264.7) was purchased from National Cell Bank of Iran (NCBI, Tehran, Iran). The cells were cultured in 75-cm2 culture flask containing DMEM medium supplemented with 15% (v/v) FBS (Gibco, USA) with antibiotics under a humidified 5% CO2 atmosphere. The cells with confluency of 80%–85%, were collected using PBS-EDTA (0.5mM) and scraping. After that, 8×105 cells were cultured on 6-well plates by using DMEM and incubated at 37 °C with 5% CO2 for 24h.

### Parasite culture and in vitro infections

The Iranian *L. major* strain (MRHO/IR/75/ER) was provided from Leishmaniasis Lab., Tehran University of Medical Sciences, Tehran, Iran. *Leishmania* promastigotes were maintained in Novy-Nicolle-Mac Neal (NNN) and incubated at 25 ºC. After that, the active and motile promastigotes were transferred into RPMI 1640 medium (Biosera, UK) supplemented with 2mM L-glutamine, 25 mM Hepes, 10% (v/v) FBS and 100 U/mL penicillin, 100 μg/mL streptomycin, and incubated at 25 °C until their population increased to use for *in vitro* assays. Promastigotes were used at the stationary phase of growth (the 5^th^ or 6^th^ day of culture) for treatment of macrophages. Primary and RAW 264.7 macrophages were infected with the stationary-phase promastigotes of *L. major* (based on parasite shape, highly motile, and flagellum length) at a ratio of 10 parasites per macrophage in different time-points (6, 12, 24 and 48 h) and incubated at 37 °C and 5% CO_2_ for further analysis. Moreover, non-infected macrophages were used at the same time points as control. In course of infection, the morphology of parasite and macrophages were investigated using invert microscopy. Moreover, the standard Giemsa staining was used for observation of intracellular parasites.

### RNA isolation, reverse transcription, and quantitative Real time-PCR analysis

Infected and uninfected macrophages were harvested from each time point by using 0.5 mM EDTA in 1x PBS and scraping. After centrifugation of the cells for 5 min at 400 × g*,* total RNA was extracted using the miRneasy® Mini kit (QIAGEN, Hilden, Germany), according to the manufacturer’s instructions. The quality and concentration of extracted RNA were determined using NanoDrop (BioTek-Synergy/HTX-Multi-mode reader). Total RNA was used for Complementary DNA synthesis with a First Strand cDNA synthesis kit (Thermo Scientific ™) following manufacturer’s instructions. It is worth noting, the miRNA-specific stem-loop primers (Zist Rouyesh, Tehran, Iran) and random hexamer primer (Yekta Tajhiz Azma, Tehran, Iran) were used for cDNA synthesis. Quantitative real-time PCR (qPCR) was performed in duplicate for each sample with ABI StepOnePlus™ (Applied Biosystems, USA) using the SYBR^®^ Green master mix Kit™ (Ampliqon, Denmark). Primers sequences are shown in Table 1. Each reaction was performed in a final volume of 20 μL containing 2 μL of cDNA (<100 ng), 10 μL of 2 × Real Q Plus Master Mix Green-high ROX (Ampliqon, Denmark), 0.4 μM of each primer (10pm), and 7.2 μL of double-distilled water. The condition of RT-qPCR was as follow one initial cycle at 95 °C for 15 min, and 40 cycles at 95 °C for 20 sec and 60 °C for 60 sec. Melting curve analysis was also performed from 55 to 95 °C with 1 °C increment in temperature. Expression levels of miRNA and target gene were normalized to mmu-SNORD-234 and mmu-β2M expression (as the reference genes) respectively. Finally, the cycling threshold was calculated and data were analyzed using 2^−ΔΔCt^ method as previously described (33).

**Table 1:** Primers sequences used for miRNAs and genes expression analysis

***A: miRNAs primers***	
miRNA name	Sequence of primer (5′-3′)
miR-24-3p	F: CGTGGCTCAGTTCAGCAG
mmu-Snord-234	F: ATCTAAGTGATTTAACAAAAATTCGTCACTAC
	Reverse universal: GAGCAGGGTCCGAGGT
B: Genes primers	
Gene name	Sequence of primer (5′-3′)
Caspase 3	F: TAAGAACTTCCATAAGAGCACTGG
	R: GCTATGATCTTCCTTAGAAACACTATCC
mmu-β2m	F: TATACTCACGCCACCCACC
	R: TCTCGATCCCAGTAGACGG

### Statistical analysis

The data were analyzed by using REST® 2009 and GraphPad Prism 5 software (San Diego, CA, USA). The difference between groups was assessed by student’s *t-test* and one-way ANOVA. All data are presented as mean ± SEM, and a *P*<0.05 was considered indicative of a significant difference.

## Results

### Bioinformatics prediction

Since previous studies have been indicated that caspase 3 and 7 are effector molecules in apoptosis (34, 35), the caspase 3 selected as target for miRNA interaction prediction. miR-vestigator, miRWalk, and DIANA algorithms were used first to find miRNA that targets 3′-UTR of caspase 3 gene. Then, further studies were performed with both literature review and related microarray analysis. Finally, miR-24-3p as anti-apoptotic miRNA was selected for expression profile analysis and further studies.

### Cell culture and L. major infection

To investigate altered miRNA and target gene, cultured macrophages (primary and cell line) were infected with stationary-phase promastigotes of *L. major*. Morphology of the intracellular parasite and infected macrophages were examined microscopically to determine the extent of the infection. Results showed successfully parasites entrance and their replication in these cells.

### miRNA and target gene expression during L. major infection

**Fig. 1:**
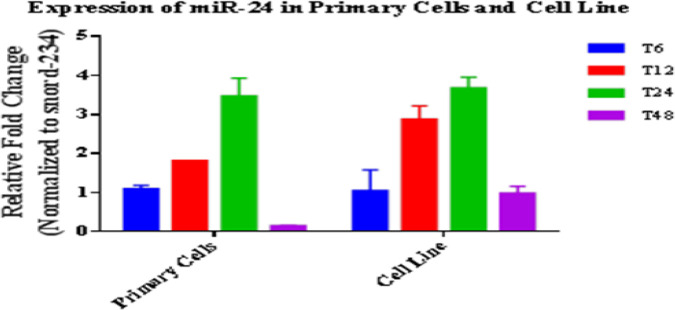
miR-24-3p expression profile in infected primary and cell line macrophages at 6, 12, 24 and 48 hours post-infection relatively to non-infected cells. Results were expressed using the 2^−ΔΔCt^ method. Data are presented as mean ± SEM. Each experiment was performed in duplicate and columns represent their data

**Fig. 2:**
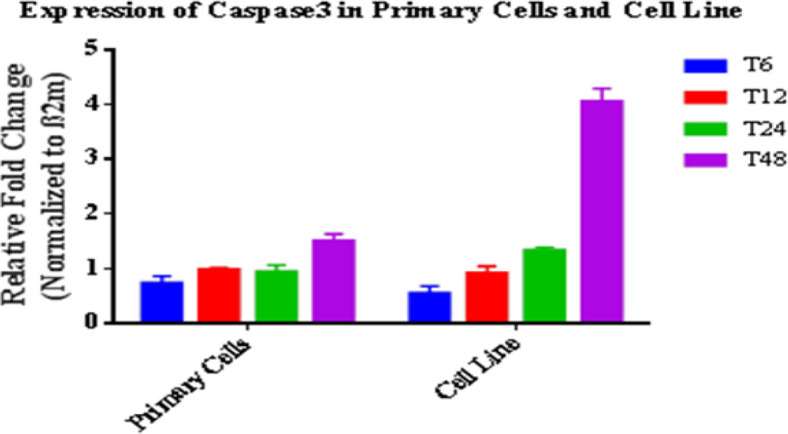
Caspase 3 expression profile in infected primary and cell line macrophages at 6, 12, 24 and 48 hours’ post-infection relatively to non-infected cells. Results were expressed using the 2^−ΔΔCt^ method. Data are presented as mean ± SEM. Each experiment was performed in duplicate and columns represent their data

Expression changes of miRNA and target gene in macrophages (primary and cell line) infected with *L. major* were investigated at indicated times post-infection. Number of infected cells and parasite load were inspected microscopically. RT-qPCR results indicated that the miRNA and gene expression profiles changed after infection. In fact, during parasite entrance and replication, the parasite dysregulated the miRNA and target gene expression. In primary cells, expression of anti-apoptotic miR-24-3p increased at the first hours especially at 12 and 24 h (1.8 and 3.4 folds with *P*<0.05, respectively) and decreased at 48 h after infection. Increase in the expression levels of miR-24-3p was significant from 6 to 24 h (Fig. 1). Indeed, parasite changes the level of expression in the first hours in favor of its survival. Expression of caspase 3 as the effector enzyme of apoptosis decreased in the first hours after infection (*P*<0.05). However, its expression increased 48 h after *L. major* infection (1.49 fold, *P*<0.05). In cell line, expression of anti-apoptotic miR-24-3p increased at the first hours especially at 12 and 24 h (2.84 and 3.68 folds with *P*<0.05, respectively) and decreased at 48 h after infection (0.95 fold, *P*<0.05) (Fig. 2). Expression of caspase 3 significantly decreased in the first hours after infection (0.4 fold, *P*<0.05), but its expression significantly increased after more time passed when the cells entered apoptosis phase (4.0 fold, *P*<0.05). Notably, comparison of the miRNA and its target gene expression profiles showed a similarity pattern in two cells relatively to non-infected cells (Figs. 1, 2).

## Discussion

According to WHO, Leishmaniasis is considered as neglected tropical and category 1 disease (5). Moreover, despite the advancements in diagnosis and therapy of the disease, there is no satisfactory treatment for leishmaniasis and the disease is still a serious public health problem in several countries (36, 37) including Iran (38). Therefore, developing novel therapeutic approaches for control and treatment of the disease are urgent need.

In recent years, miRNAs discovery has been one of the most important advancements in molecular biology (24, 25). Indeed, Rapid progression of miRNA-related research has been revealing the potential of miRNAs as novel diagnostic and treatment tool for many of diseases such as parasitic diseases. Various studies have documented the importance of miRNAs in the regulation of physiological and pathological processes in parasites (39–41).

Our study showed that *L*. *major* infection alters macrophage miRNA and its target gene expression profile in different time points. We first predicted miRNAs targeting 3′UTR of target gene mRNA using different databases. Based on the results and given the importance of miRNAs in regulation of apoptotic pathways, anti-apoptotic miR-24-3p was selected. The expression miRNA and target gene were evaluated by qRT-PCR method. Our results determined that their expression were significantly different in course of infection compared with uninfected cells. Nowadays, using high-throughput methods such as Next Generation Sequencing (NGS) and microarray, parasitic infection can change the miRNAs expression profile of macrophages and dendritic cells (26–28, 42, 43). However, they are high-cost techniques not accessible in all laboratories. Instead of these techniques, we applied quantitative Real-time PCR which can provide throughout information about miRNAs and genes expression and their changes during parasitic infections.

To date, intracellular parasites such as *Toxoplasma gondii* (44), *L. major* (26), and *L. donovani* (27) can change miRNAs expression profile of the host cells. Overall, 64 out of 365 studied miRNAs were dysregulated. The parasite changes miRNA expression profile of cells in favor to its survival (26). The expression of 85 out of 940 identified miRNAs has been altered after *L. donovani* infected RAW264.7 mice macrophages (27). *L*. *amazonensis* infection changes miRNA profile of murine macrophage (31). Our results are in line with mentioned studies emphasizing on the changes in miRNA profiles after *Leishmania* infection. Moreover, we also compared the miRNA expression profile in primary and RAW264.7 macrophages with focusing on miRNA regulatory role in apoptosis. Comparing miRNAs expression profile of host and parasite in infected and uninfected cells can provide a better understanding of miRNAs expression changes during infection. In comparing miRNA and gene expression patterns, similarity was observed in two cells. Therefore, this work suggests that cell line can be a good pattern of expression changes during parasitic infections, since application of peritoneal macrophages confronts several problems including: cells sensitivity in culture medium, difficulty in isolation of the cells and ethics for animal death. Moreover, cell line can use as a pilot in vivo assays.

According to our results, *L. major* infection induces increase in miR-24-3p as anti-apoptotic in the first hours post-infection. This miRNA can interact and regulate caspase 3 gene to expand the life time of macrophage and stabilize parasite in the cells. Therefore, antagomir-24-3p has the therapeutic potential for *L. major* infection control and treatment. Anti-apoptotic and pro-apoptotic miRNAs target pro-apoptotic and anti-apoptotic genes, respectively. For example, miR-15a and miR-16-1 induce apoptosis in chronic lymphoblastic leukemia (CLL) by targeting anti-apoptotic factors such as Bcl2 and BAD (45), while miR-155 inhibits apoptosis by targeting Fas-associated death domain-containing protein (FADD), caspase 3 and caspase 7 (46). Overall, these studies indicate the potential of different *Leishmania* species for changing miRNAs expression in host cells. However, more investigations are needed to understand the biological importance of changed miRNAs in host cells.

## Conclusion

*Leishmania* uses different mechanism to survival in macrophages like apoptosis. The result showed that *L. major* alters miR-24a-3p expression as anti-apoptotic miRNA in macrophages in different time points and inhibits pro-apoptotic genes (Caspases 3). Therefore, usage of antagomir-24-3p may have the therapeutic potential for control and treatment of leishmaniasis. Besides that, this work suggests that cell lines can be a good pattern of expression changes during parasitic infections, due to difficulty of peritoneal macrophages application.
